# Intestinal Obstruction in the Third Trimester of Pregnancy: Maternal and Fetal Outcomes

**DOI:** 10.7759/cureus.84980

**Published:** 2025-05-28

**Authors:** Shamail A Syed, Fatima Latif, Amna Rafique, Asila Anwar

**Affiliations:** 1 Obstetrics and Gynaecology, Maroof International Hospital, Islamabad, PAK; 2 Anesthesia and Critical Care, Shifa International Hospital Islamabad, Islamabad, PAK; 3 General Practice, Mersey and West Lancashire Teaching Hospital, Shrewsbury, GBR

**Keywords:** adverse pregnancy-fetal outcomes, complications in pregnancy, intrauterine growth restriction (iugr), laparoscopy in pregnancy, laparotomy, mri, small-bowel obstruction, surgical emergencies, third trimester complications, total parenteral nutrition (tpn)

## Abstract

Small bowel obstruction (SBO) is a rare yet life-threatening event in pregnancy. If not diagnosed and treated promptly, it can lead to an increase in maternal as well as foetal morbidity and mortality. In the majority of cases, small bowel obstruction is caused by adhesions from previous pelvic surgeries. We herein report a case of SBO in a 28-week pregnant woman, who had a previous history of laparotomy for ectopic pregnancy. She presented with severe epigastric pain and vomiting in the emergency department. On initial examination, the patient was clinically stable but with a distended abdomen. Abdominal ultrasound showed fluid-filled dilated small bowel loops, but was otherwise unremarkable. Conservative treatment was provided, and she was kept on nothing by mouth. Despite intravenous pain killers and antiemetics, the symptoms remained unsettled 72 hours after admission. A clinical suspicion of SBO was made, and an urgent MRI was carried out, suggesting small bowel obstruction. She underwent an immediate laparotomy. Intraoperatively, bands of omentum were found to be adherent to the distal ileum. The adhesions were resected, and the affected ileum showed no signs of ischaemia, so it was preserved. Her postoperative course was uneventful. She later had a caesarean section for intrauterine growth restriction at 37 weeks and delivered a 2.1 kg baby.

Diagnosis of SBO is difficult, especially in the second and third trimesters, as the symptoms often are mistakenly attributed to the pregnancy itself. There is a reluctance in getting investigations such as a CT scan done due to the risk of exposure of the foetus to ionising radiation which can lead to a delay in diagnosis and treatment. MRI can safely be used in pregnancy as a modality to diagnose intestinal obstruction and to determine the aetiology. Once diagnosed, the optimal management mainly depends on the aetiology and the gestational age.

## Introduction

Intestinal obstruction in pregnancy is an uncommon and serious non-obstetrical surgical condition that may be associated with significant maternal as well as foetal morbidity and mortality, if not managed promptly [1]. The incidence of intestinal obstruction during pregnancy is estimated at 1:1500-1:66431 pregnancies and is more commonly seen in the second and third trimesters of pregnancy [2]. Early diagnosis and successful treatment are paramount in reducing significant mortality associated with the condition. The large intestine is more commonly involved, especially the sigmoid colon and cecum [2]. The condition is often met with diagnostic and therapeutic challenges due to the rarity of occurrence, overlapping symptomatology, concerns over radiological evaluation, and risks involved with surgery and anaesthesia [1]. Symptoms include abdominal pain (98%), vomiting (82%), and constipation (30%) [2]. 

The most common imaging examination in the diagnosis of intestinal obstruction is the abdominal X-ray, which is positive in 82% of pregnant women, and a computed tomography (CT) scan; however, it has harmful effects of ionising radiation on the foetus [2]. Ultrasound and magnetic resonance imaging(MRI) are considered safe and can provide useful information in diagnosing intestinal obstruction; often, intravenous contrast is not needed [3]. In the absence of signs of peritonitis, and when adhesions are suspected to be the cause of obstruction, conservative treatment and hyperalimentation may improve the maternal and foetal outcome [3]. Surgery is needed in the majority of cases when there are signs of impending bowel strangulation pain [2]. The overall prognosis is poor during the second and third trimesters; the foetal mortality rate reaches up to 36% in the second trimester and 64% in the third trimester [2], while the risk of maternal death is 6-20% [3,4]. The most important cause of intestinal obstruction in pregnant women is adhesions due to previous surgeries (54.6%); others include intestinal torsion (25%), colorectal cancer (3.7%), hernia (1.4%), and others (10%) [2]. Adhesive obstruction occurs more frequently in advanced pregnancy, 61% in the second, and 28 % in the third trimester, respectively [5].

We present herein a case of small bowel obstruction at 28 weeks of pregnancy, for which a diagnosis was made based on clinical suspicion and MRI findings. She underwent a laparotomy, and adhesions were found to be the cause of obstruction, attached to the distal ileum. The adhesive bands were removed, and the ileal loop was released and preserved. She later had a caesarean section due to foetal growth restriction at 37 weeks of pregnancy.

## Case presentation

A 36-year-old woman (Gravida 5, Para 3, Abortion 1) was admitted to the obstetrics unit through emergency at 28 weeks and 2 days of pregnancy with severe epigastric pain and vomiting. She had a previous history of laparotomy for ectopic pregnancy, with only one alive and healthy 13-year-old son. She also had a history of one neonatal death and an infant death due to glycogen storage disease. All were delivered vaginally. It's important to note that she did not have any previous history of intestinal diseases or obstruction. She described the pain as colicky, with severe nausea and dry retching. She was passing flatus and had moved her bowels normally the day prior. She denied any urinary symptoms.

**Figure 1:**
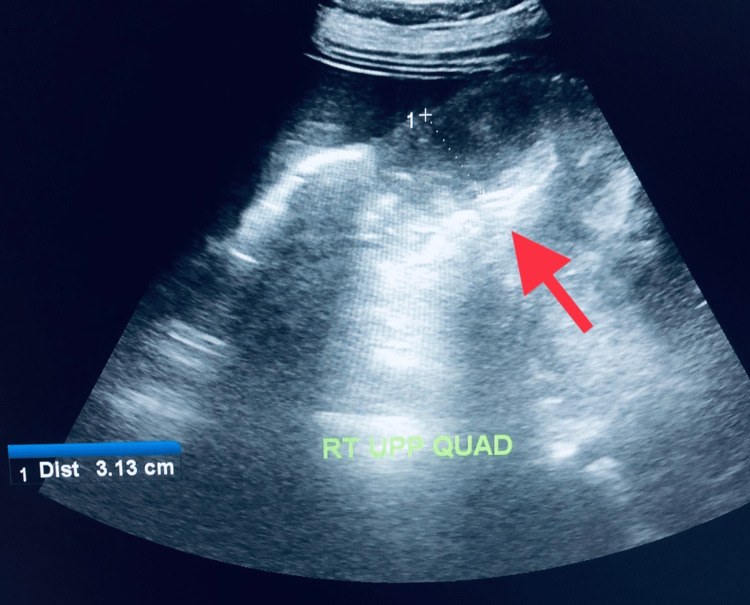
Ultrasound of the abdomen The image shows dilated small bowel loops with a diameter of more than 3 cm (red arrow).

At presentation, she was afebrile, with a normal heart rate and blood pressure. Her abdomen was distended without tenderness, and the bowel sounds were reported to be normal. The foetal heart sounds were also normal. She had a normal haemoglobin of 11 g/dl, a leucocyte count of 12.540 x 10 ^9^/l, and a C-reactive protein (CRP) level of 11.5 mg/dl. A urine routine examination showed 3+ ketones and no protein. Her liver function tests, renal function tests, serum amylase, and lipase were within normal limits. Initial serum electrolytes were normal, but she later developed hypokalemia, with potassium levels dropping to 2.7 mEq/L, due to unresolved vomiting. Fetal ultrasound showed a 28-week healthy foetus with normal umbilical doppler indices and liquor volume. The abdominal ultrasound showed multiple fluid-filled dilated loops of small bowel with hyperperistalsis in the left abdomen, and a suspicion of acute/subacute intestinal obstruction was given (Figure 1).

**Figure 2:**
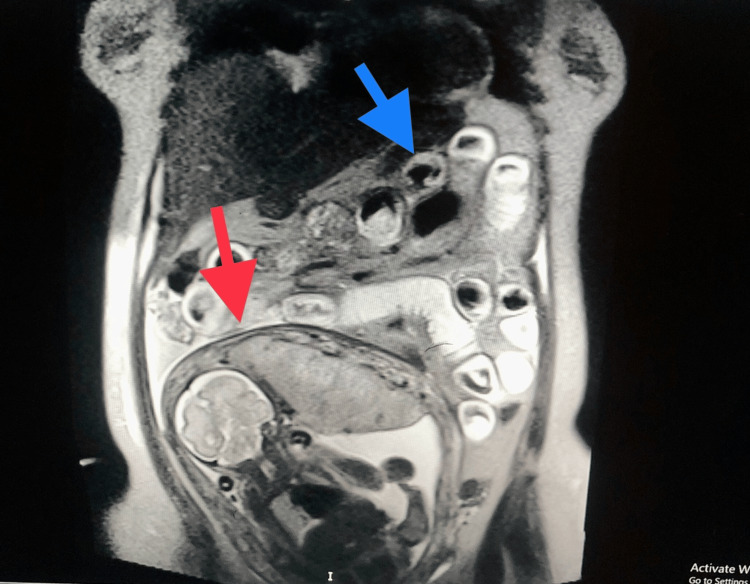
MRI scan The MRI scan image confirms small bowel obstruction. Dilated small bowel loops were seen in the upper quadrants (blue arrow). The fetus is seen in utero (red arrow).

A multidisciplinary team was involved in the care, which included a gastroenterologist, general surgeon, neonatologist, anaesthetist, and obstetrician. For the next 72 hours, she remained on conservative management. She was kept on nothing by mouth (nil per os) and was given intravenous fluids, antiemetics, antispasmodics, antibiotics, analgesics, and proton pump inhibitors; however, the pain and vomiting gradually worsened. A suspicion of bowel obstruction was made, and an urgent MRI was carried out, which confirmed small bowel obstruction (Figure 2).

**Figure 3:**
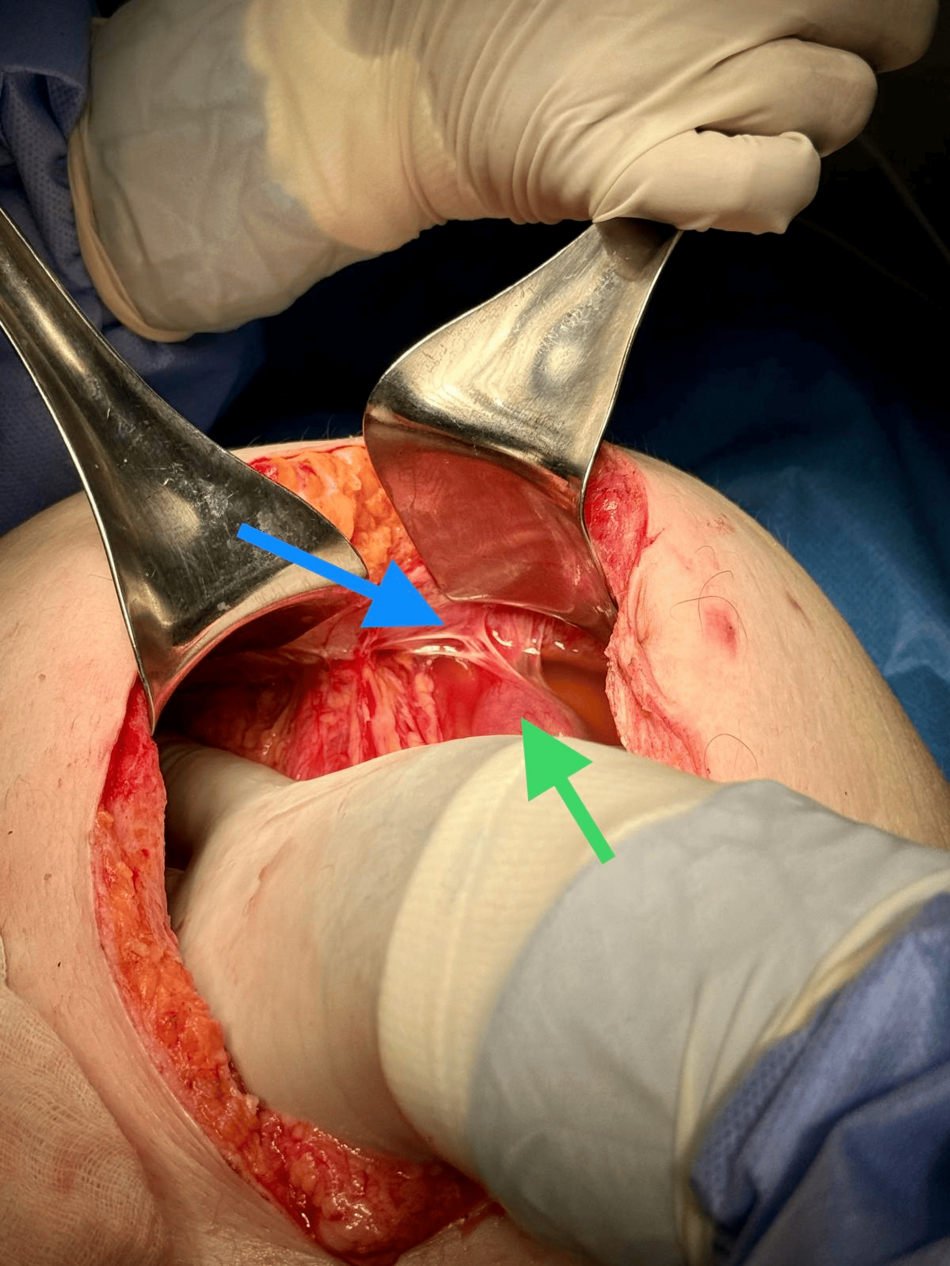
Intraoperative image Adhesions (blue arrow) are seen attached to a segment of the ileum (green arrow).

She eventually underwent laparotomy on the 3rd day of hospitalisation, after completing the dosage of dexamethasone for foetal lung maturity and receiving intravaginal progesterone pessaries to prevent premature birth. Intra-operative assessment showed adhesive bands attached to the distal ileum, which were released (Figure 3).

**Figure 4:**
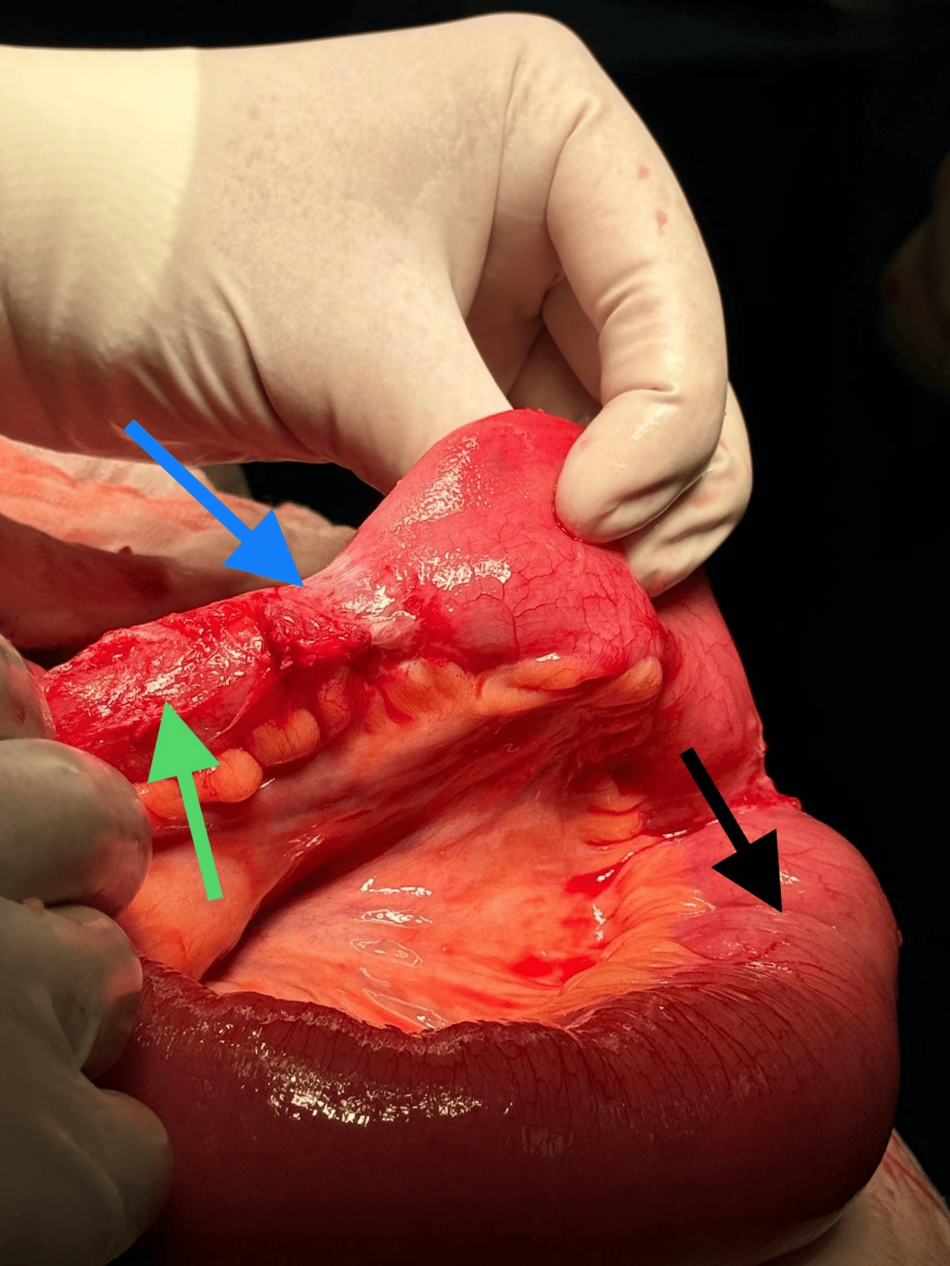
Intraoperative image showing a healthy segment of the ileum Healthy segment of the ileum (green arrow) freed from the point of attachment of adhesions shown (blue arrow). The cecum is shown with the black arrow.

The affected segment of the ileum was preserved, as it showed no signs of ischaemia, and normal peristaltic movements were observed (Figure 4).

Postoperatively, she made an uneventful recovery and was discharged on the 8th day of admission. She subsequently underwent an emergency Caesarean section at 37 weeks of gestation due to reduced foetal movements, foetal growth restriction with deranged foetal doppler indices, and oligohydroamnios. The birth weight of the baby was 2.1 kg. Following birth, both the mother and the baby made an unremarkable recovery.

## Discussion

A high index of clinical suspicion coupled with timely surgical intervention increases the chances for a favourable outcome in situations where intestinal obstruction complicates a pregnancy; it is very important to note that both the mother and the foetus are at risk [1]. SBO can broadly be categorised into simple obstructions, which involve mechanical blockage without ischaemia, and strangulated obstructions, which are associated with compromised blood flow, bowel ischaemia, and potential necrosis [6].

Postoperative adhesions are the commonest cause of SBO; however, even without prior abdominal surgeries, adhesions cannot be ruled out, as 11% of adhesions are congenital [6]. A lot of symptoms of SBO overlap with pregnancy itself, like abdominal pain and vomiting, which can be confused with preterm labour and hyperemesis gravidarum. Physical examination is often challenging as the gravid uterus poses limitations to proper examination of the abdominal quadrants [5]. Constant nonremitting abdominal pain along with excessive vomiting persisting into the second and third trimesters of pregnancy should warrant suspicion of intestinal obstruction. Similarly, intermittent colicky pain of SBO can be mistaken for preterm labour pain. The signs of fever, tachycardia, tachypnoea, and hypotension usually manifest later on, unfortunately, when severe acidosis and infection manifest [6]. 

Patients with suspicion of SBO should be managed on an individual basis with a multidisciplinary approach [7]. It is also vital to involve the neonatology team as the risk of preterm delivery is up to 45% in these women [8]. The delay from admission to diagnosis and management continues to be a significant cause of morbidity and mortality [9]. A crucial aspect in management is to differentiate whether there is actual or impending small bowel ischemia and therefore the need for urgent surgery [10]. Prompt diagnoses are critical to prevent severe complications, including gangrenous bowel, perforations, and sepsis [11]. Laboratory investigations are of little use; the most common findings are electrolyte imbalance and deranged renal function tests due to severe dehydration. Leucocytosis is common in pregnancy; however, an increasing trend should alert obstetricians to causes that are more serious [12]. Even though there is significant maternal and foetal mortality associated with acute abdominal emergencies, the apprehension about exposing the foetus to radiation with investigations like X-rays and CT scans still exists. Foetal risk of anomalies, abortion, or growth restriction has not been reported with radiation exposure of less than 50 mGy [5]. Ultrasound is not the most sensitive modality to rule out SBO, especially in pregnant women; a study showed 55% of ultrasound findings similar to surgical findings in the non-pregnant population [13]. A CT scan has been recommended by the American College of Radiology as the initial imaging modality for evaluating intestinal obstruction in non-pregnant patients with clinical suspicion [5]. With the widespread availability of MRI, it has become a safer and more sensitive radiological investigation to be used to diagnose SBO and confirm the aetiology of the condition. 

Surgical management is indicated when there are signs of bowel ischaemia, foetal distress [5], or the cause of SBO is volvulus or internal hernias [8]. The surgical techniques in pregnant women do not differ from those in the general population [6]. Once the decision of surgery is made, the surgical approach (laparoscopy vs laparotomy) depends on the expertise and equipment availability. It has been shown that laparoscopy can be performed safely during any trimester of pregnancy with minimal morbidity to the foetus and mother; however, most still undergo laparotomy, especially if complicated or there is diagnostic uncertainty [13]. The management of the foetus depends on the gestational age; unless the pregnancy is full term and ready to be delivered, minimum handling of the uterus should be carried out by the surgeons as much as possible. In one review, only 21 % of the foetuses were delivered during the laparotomy [6].

Careful selection of patients for conservative management should be considered with a low threshold for laparotomy, as a delayed intervention can often lead to serious consequences, and the majority of the patients will still require surgical exploration because the common causes, such as adhesions, will persist, and there is always a risk of symptomatic recurrence [7]. With MRI showing no concerning features of ischaemia and strangulation and a normal fetal ultrasound and heart tracing, a trial of conservative management can be started [14]. Conservative approaches include nasogastric decompression, intravenous fluid, and intravenous antispasmodics [5].

SBO can cause malnutrition in the mother, hence increasing the risk of spontaneous abortion, congenital malformations, intrauterine growth restriction (IUGR), preterm delivery, and perinatal morbidity and mortality [15]. Total parenteral nutrition (TPN) is known to provide adequate nutrition in patients undergoing conservative treatment for SBO, provided there is vigilant foetal and maternal monitoring, but unfortunately, it is costly. 

Over the years, there has been an increase in the maternal survival rates in patients with SBO, which is likely due to early diagnosis and timely intervention. Compared to the maternal mortality rate, which is reported to be 6%, the foetal mortality rate is higher at 26%. Therefore, it is vital not to delay the use of modalities like MRI in pregnant women to confirm the diagnosis of SBO and immediately initiate management, whether it is conservative, which includes initiation of TPN, or surgical intervention, because as the mother's condition deteriorates, the foetal condition worsens more rapidly.

## Conclusions

When SBO is suspected in a pregnancy, only through urgent intervention can the morbidity and mortality be decreased. A multidisciplinary team approach is highly recommended to safeguard a sufficient standard of care for both the mother and the baby. With medical advancements and MRI being freely available and safe in pregnancy, there should be no reason why the diagnosis is missed or the treatment delayed.
